# Clinically-evident tophi are associated with reduced muscle force in the foot and ankle in people with gout: a cross-sectional study

**DOI:** 10.1186/s13047-017-0207-4

**Published:** 2017-06-19

**Authors:** Sarah Stewart, Nicola Dalbeth, Simon Otter, Peter Gow, Sunil Kumar, Keith Rome

**Affiliations:** 1 Department of Podiatry, Health & Rehabilitation Research Institute, Auckland University of Technology, Private Bag 92006, Auckland, 1142 New Zealand; 2 Faculty of Medical and Health Sciences, The University of Auckland, Private Bag 92019, Auckland, 1142 New Zealand; 3 Department of Rheumatology, Auckland District Health Board, P.O. Box 92189, Auckland, New Zealand; 4 School of Health Sciences, University of Brighton, 49 Darley Road, Eastbournem, Brighton BN20 7UR UK; 5 Department of Rheumatology, Counties Manukau District Health Board, Auckland, New Zealand

**Keywords:** Gout, Foot, Dynamometry, Tophi

## Abstract

**Background:**

The foot and ankle represent a common site for tophi in people with gout, yet it is unclear whether the presence of tophi is related to impaired muscle function. This study aimed to determine the association between foot and ankle tophi and muscle force in people with gout.

**Methods:**

Participants with gout were stratified into two groups based on the presence of clinically-evident tophi affecting the foot or ankle on physical examination. Isometric muscle force for plantarflexion, dorsiflexion, inversion and eversion was measured using static dynamometry. Mixed-models regression was used to determine the difference in muscle force between the two groups while adjusting for age, disease duration and foot pain. This model was also used to determine the difference in muscle force between presence and absence of tophi at specific locations within the foot and ankle. In addition, Pearson’s correlations were used to determine the association between total foot tophus count and muscle force.

**Results:**

Fifty-seven participants were included (22 with foot or ankle tophi and 35 without foot or ankle tophi). Foot and ankle tophi were most often seen at the Achilles tendon. After adjusting for age, disease duration and foot pain, participants with tophi had significantly reduced muscle force during plantarflexion (*P* < 0.001), dorsiflexion (*P* = 0.003), inversion (*P* = 0.003) and eversion (*P* = 0.001) when compared to participants without tophi. Those with Achilles tophi had significantly reduced force during plantarflexion (*P* < 0.001), inversion (*P* = 0.008) and eversion (*P* = 0.001). No significant differences in muscle force were observed between the presence and absence of tophi at other foot or ankle locations. There were also no significant correlations between total foot tophus count and muscle force (all *P* > 0.05).

**Conclusion:**

In people with gout, clinically-evident foot or ankle tophi are associated with muscle force deficits during foot plantarflexion, dorsiflexion, inversion and eversion, which persist despite adjusting for age, disease duration and foot pain. Tophi at the Achilles tendon, which associate with force deficits, may contribute to reduced muscular activation and consequent disuse muscle atrophy.

## Background

Gout is a common form of arthritis resulting from the deposition of monosodium urate (MSU) crystals in structures of the musculoskeletal system [1–3]. In the absence of urate-lowering therapy, tophi develop in some people with long-standing gout [4, 5], and result from accumulation of MSU crystals and a chronic inflammatory granulomatous tissue response [6]. Tophi form within subcutaneous tissues, joints and tendons and contribute to clinical features of chronic arthropathy, including inflammation, pain and joint effusion [7, 8]. Intra-articular tophi have been associated with limitations in function and mechanics of joints in both the upper limb [9] and knees in people with gout [10]. The functional impact of tophi has also been reported to limit the ability of people with gout to participate in normal daily activities [11].

The foot and ankle regions represent a major site for tophus deposition [12–15] and people with gout experience persistent pain and disability related to the foot [16, 17]. Lower limb, foot and ankle biomechanical studies have shown walking impairments [18–20] and a loss of normal foot joint function in people with gout [21]. Furthermore, foot and ankle muscle force in people with gout is significantly reduced compared to people without gout [22]. Foot and ankle muscle strength plays a fundamental role in the ability to carry out functional activities, including walking. Despite the prominent foot-related disability and impairment observed in people with tophaceous gout, the relationship between tophus presence and muscle force in the foot has not been yet been assessed.

Despite the frequent prevalence of tophus deposition within the foot and ankle [12–14], it is unclear whether tophus presence is associated with local impairments in foot and ankle muscle strength in people with gout. The aim of this study was to determine the association between clinically-evident tophi and muscle force in the foot and ankle in people with gout.

## Methods

Participants with gout were recruited from the rheumatology department at Counties Manukau District Health Board, Auckland, New Zealand. All participants fulfilled the 1977 preliminary American Rheumatism Association gout classification criteria [23]. Participants were excluded if they were <20 years old, had undergone foot and/or ankle surgery in the previous 3 months, had a history of other inflammatory arthritis, were unable to walk 10 m unaided, or were experiencing an episode of acute arthritis at the time of the clinical visit. The study was approved by the Auckland University Ethics Committee (14/57). All participants provided written informed consent prior to data collection.

Participants were stratified into two groups based on the presence or absence of at least one clinically-evident tophi in either the right or left foot or ankle region. Assessment for tophi was undertaken by a single podiatric researcher with experience in the clinical assessment of gout (SO). Tophi were defined as palpable nodules evident within periarticular or subcutaneous tissue, which is a simple and feasible method for tophus assessment [24]. The location of tophi in each foot was also recorded.

Demographic information including age, sex, ethnicity, body mass index (BMI), current medications and co-morbidities was recorded for all participants. Gout disease characteristics, including disease duration and flare history were also recorded. Patient-reported foot pain and global pain over the past week were assessed using 100 mm Visual Analog Scales (VAS) [25]. Foot-related pain and disability was measured using the Manchester Foot Pain and Disability Index [26].

Isometric muscle force for ankle plantarflexion, dorsiflexion, inversion and eversion was measured using a CITEC hand-held dynamometer (CIT Technics, Haren, Netherlands). Hand-held dynamometry provides an easy-to-use tool for the objective quantification of muscle strength in clinical settings and has been shown to be reliable for assessment of foot and ankle muscles [27]. The device is calibrated to a sensitivity of 0.1% and a range of 0 to 500 N (N). Participants were positioned seated during testing with hips flexed and knees extended. The examiner stabilised the lower leg and foot proximal to the ankle joint with the foot and ankle maintained in a neutral position. Three consecutive contractions of three to 5 seconds were performed for each muscle group and the maximum force for each contraction was recorded. The ‘make’ technique, in which the examiner held the dynamometer stationary while the participant exerted maximal external force against it [27]. The dynamometer was positioned on the plantar aspect of the foot proximal to the metatarsophalangeal joints for plantarflexion; on the dorsum of the foot proximal to the metatarsophalangeal joints for dorsiflexion; on the medial aspect of the first metatarsal shaft for inversion; and on the lateral aspect of the fifth metatarsal shaft for eversion. The mean of the three measurements for each right and left limbs were used in the analysis.

Data were analysed using SPSS version 20.0 (IBM Corp., Armonk, NY). Measures between participants with foot and/or ankle tophi and those without foot and/or ankle tophi were compared using mixed linear regression models. A participant-specific and participant-nested random effect for foot-side was included in the model to account for the repeated measures in the right and left feet for each individual participant. All analyses were adjusted for age, disease duration and foot pain VAS. To account for multiplicity, a Bonferroni adjusted alpha of *P* < 0.0125 was used when analysing the four muscle groups. The same mixed linear regression model was also used to determine the difference in muscle force between participants with and without foot or ankle tophus at each of the following seven sites: hallux, lesser toes, first metatarsophalangeal joint (1MTP), lesser metatarsophalangeal joints (MTPs), midfoot, heel and Achilles tendon. In addition, the correlation between total number of clinically-evident tophi at the foot and/or ankle and the muscle force outcomes were assessed using Pearson’s correlation testing.

## Results

**Fig. 1:**
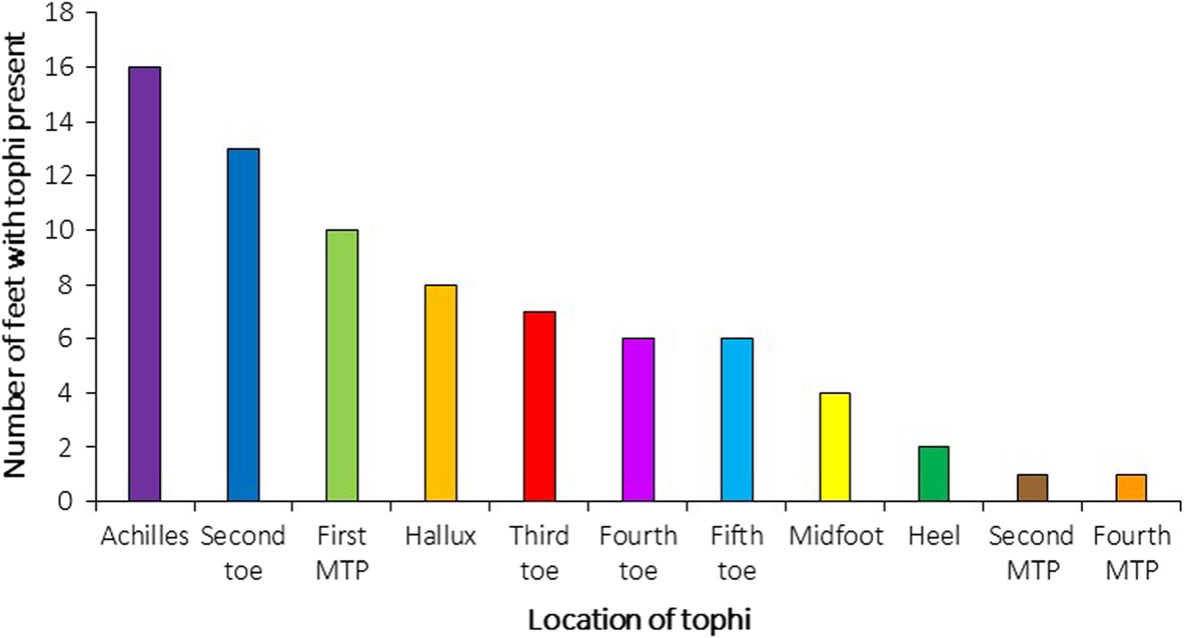
Location of tophi in participants with foot or ankle tophi (*n* = 44 ft). MTP: metatarsophalangeal joint

A total of 57 participants were included in the study (Table 1). Thirty-five participants had no clinical evidence of tophi in the foot or ankles, and 22 had clinical evidence of foot and ankle tophi. In those with foot or ankle tophi present, the mean (SD) foot tophus count was 4.6 (5.6). The most common area for tophi in this group was the Achilles tendon (*n* = 16 tendons, 36%) (Fig. 1). Of those participants with Achilles tendon tophi, all had tophi present in at least one other location in the body. Participants in both groups were primarily middle-aged men with frequent comorbidities, including hypertension, cardiovascular disease and diabetes. Participants with foot tophi had a significantly longer gout disease duration compared to the group with no foot tophi present (*P* < 0.001) and had a significantly higher overall body tophus count (*P* = 0.001). Participants with foot tophi also reported significantly greater foot pain and disability scores (MFPDI) compared to those with no foot tophi (*P* = 0.044).

**Table 1:** Participant characteristics

	Foot or ankle tophi		*P*
	Absent	Present	
N	35	22	
Male, *n* (%)	31 (89%)	19 (86%)	0.81
Age, years	60 (14)	60 (12)	0.89
BMI, kg/m^2^	33 (8)	33 (6)	0.68
Ethnicity			
European, *n* (%)	11 (31%)	6 (27%)	0.26
Māori, *n* (%)	8 (23%)	3 (14%)	
Pacific, *n* (%)	13 (37%)	13 (59%)	
Asian, *n* (%)	3 (9%)	0 (0%)	
Type 2 diabetes, *n* (%)	14 (40%)	7 (32%)	0.53
Stage 3 or worse chronic kidney disease, *n* (%)^a^	19 (54%)	17 (77%)	0.06
Hypertension, *n* (%)	26 (74%)	16 (73%)	0.90
Cardiovascular disease, *n* (%)	12 (34%)	6 (27%)	0.58
Gout disease duration, years	11 (10)	25 (13)	**< 0.001**
Gout flares in previous 3 months	0.6 (1.1)	0.5 (0.9)	0.55
Serum urate, mmol/L	0.41 (0.13)	0.39 (0.13)	0.58
Urate lowering therapy, *n* (%)	31 (89%)	21 (96%)	0.59
Total foot tophus count	0 (0)	4.6 (5.6)	**0.001**
Total body tophus count	1.7 (4.6)	12.9 (12.8)	**0.001**
Foot pain VAS, mm	5.7 (30.4)	15.5 (38.5)	0.05
Global pain VAS, mm	12.4 (27.9)	14.4 (35.5)	0.74
Manchester Foot Pain and Disability Index	10.6 (12.5)	16.6 (15.8)	**0.044**

Values are mean (SD) unless otherwise specified. ^a^Defined as estimated glomerular filtration rate (eGFR) of <60 mL/min/1.73mm^2^; ^b^For those in employment; Bolded *P* values indicate significant difference between groups at *P* < 0.05. *BMI* body mass index, *VAS* visual analogue scale

The distribution of residuals from the linear models for all muscle force variables demonstrated sufficient normality to carry out parametric testing. After adjusting for age, disease duration and pain score, participants with foot or ankle tophi had significantly reduced muscle force compared to participants without tophi during plantarflexion (75.4 N vs. 99.9 N, *P* < 0.001), dorsiflexion (55.7 N vs. 74.2 N, *P* = 0.003), inversion (45.5 N vs. 58.2 N, *P* = 0.003) and eversion (43.4 N vs. 56.2 N, *P* = 0.001) when compared to participants with no foot tophi (Table 2). The site specific analysis revealed that participants with tophi at the Achilles tendon demonstrated reduced muscle force compared to participants without Achilles tendon tophi during plantarflexion (94.2 N vs, 65.1 N, *P* < 0.001), inversion (54.9 N vs. 40.9 N, *P* = 0.008) and eversion (53.4 N vs. 37.0 N, *P* = 0.001). A similar non-significant trend was also noted with Achilles tendon tophi and dorsiflexion force (69.4 N vs. 51.2 N, *P* = 0.016) (Table 3). No differences were noted in muscle force between the presence and absence of tophi at the other foot sites (*P* > 0.05). There were no correlations between muscle force and number of tophi affecting the foot and ankle (−0.11 > *r* < 0.02, *P* > 0.26 for all analyses).

**Table 2:** Association between foot and ankle tophus presence and muscle force^a^

	Least-squares mean (SD)		Diff.	95% CI for Diff.		*P*
	Foot/ankle tophus absent	Foot/ankle tophus present		Lower	Upper	
Plantarflexion force, *N*	99.9 (31.5)	75.4 (31.0)	24.5	11.5	37.4	**<0.001**
Dorsiflexion force, *N*	74.2 (28.9)	55.7 (18.3)	18.5	6.6	30.3	**0.003**
Inversion force, *N*	58.2 (20.2)	45.5 (19.6)	12.6	4.4	20.9	**0.003**
Eversion force, *N*	56.2 (19.3)	43.4 (18.8)	12.9	5.0	20.8	**0.001**

^a^Results are presented adjusted for gout disease duration, age and foot pain VAS. Bolded *P* values indicate significant difference between groups at Bonferroni-adjusted significance level of *P* < 0.0125. *Diff.* difference in least-squares mean between the two groups, *CI* confidence interval

**Table 3:** Association between tophus location at each site in the foot and ankle and muscle force^a^

		Least-squares mean (SD)		Diff.	95% CI		*P*
	Tophus location	Absent	Present		Lower	Upper	
Plantarflexion force	Hallux	91.3 (30.2)	72.1 (29.4)	19.1	−2.6	40.9	0.083
	Lesser Toes	92.2 (30.1)	77.5 (29.4)	14.7	−0.9	30.3	0.065
	First MTP	91.0 (30.4)	77.7 (29.8)	13.4	−6.1	32.8	0.175
	Lesser MTPs	89.6 (30.5)	100.0 (29.3)	−10.3	−52.3	31.6	0.627
	Midfoot	89.4 (30.4)	101.6 (29.8)	−12.3	−42.4	17.9	0.422
	Heel	89.3 (30.3)	117.5 (28.6)	−28.2	−69.6	13.2	0.177
	Achilles	94.2 (29.1)	65.1 (29.3)	29.1	13.2	45.0	**<0.001**
Dorsiflexion force	Hallux	68.1 (26.8)	47.9 (25.8)	20.2	1.1	39.3	0.038
	Lesser Toes	67.9 (27.3)	59.5 (26.8)	8.4	−5.8	22.6	0.243
	First MTP	66.5 (27.5)	67.3 (27.0)	−0.7	−18.4	16.9	0.933
	Lesser MTPs	66.9 (27.4)	52.3 (26.3)	14.6	−23.1	52.3	0.444
	Midfoot	66.0 (27.3)	81.4 (26.8)	−15.4	−42.6	11.7	0.262
	Heel	66.1 (27.2)	94.8 (27.2)	−28.7	−68.0	10.6	0.149
	Achilles	69.4 (27.1)	51.2 (27.1)	18.2	3.4	32.9	0.016
Inversion force	Hallux	53.4 (19.1)	46.4 (18.8)	7.1	−6.8	21.0	0.314
	Lesser Toes	53.7 (19.1)	48.7 (18.4)	5.0	−4.8	14.9	0.311
	First MTP	53.0 (19.2)	52.2 (18.8)	0.8	−11.5	13.1	0.899
	Lesser MTPs	53.0 (19.1)	48.6 (18.3)	4.4	−21.9	30.6	0.742
	Midfoot	52.3 (18.9)	66.4 (18.4)	−14.1	−32.7	4.6	0.137
	Heel	52.4 (18.8)	72.4 (16.8)	−20.0	−44.4	4.4	0.106
	Achilles	54.9 (18.6)	40.9 (18.8)	14.0	3.8	24.2	**0.008**
Eversion force	Hallux	51.5 (18.2)	43.7 (17.4)	7.8	−5.1	20.8	0.233
	Lesser Toes	50.9 (18.3)	50.9 (18.0)	0.0	−9.5	9.6	0.997
	First MTP	50.9 (18.3)	50.6 (18.0)	0.3	−11.4	12.0	0.962
	Lesser MTPs	50.9 (18.3)	48.9 (17.6)	2.0	−23.2	27.2	0.875
	Midfoot	50.6 (18.2)	58.7 (17.9)	−8.2	−26.3	10.0	0.374
	Heel	50.7 (18.2)	63.2 (18.6)	−12.5	−39.5	14.4	0.355
	Achilles	53.4 (17.7)	37.0 (17.6)	16.4	6.8	26.0	**0.001**

^a^Results are presented adjusted for gout disease duration, age and foot pain VAS. Bolded *P* values indicate significant difference between groups at Bonferroni-adjusted significance level of *P* < 0.0125. *Diff.* Difference in least-squares mean between the two groups, *CI* confidence interval

## Discussion

This study has shown that in the presence of clinically-evident tophi within the foot and ankle, muscle force is significantly reduced in plantarflexion, dorsiflexion, inversion and eversion, when compared to people with gout who do not have clinical evidence of foot or ankle tophi. The analysis also suggests that tophi at the Achilles tendon may play a role in force reductions not only during plantarflexion, but also during inversion and eversion of the foot.

While it is possible that intra-muscular tophus deposition would reduce the working area of the muscle and its ability to produce adequate force [28], it seems unlikely that this is a dominant mechanism. Numerous studies have reported tophus deposition within joints, tendons and ligaments involved in foot and ankle function [12, 14, 29], but muscle deposition appears to be rare, with evidence limited to a few case reports [30]. The presence of intra-articular tophus has been shown to limit joint motion in the knee [10] and it is possible that restrictions to normal joint motion may also compromise the potential for muscles to generate force.

It has been hypothesised that the pain-avoidance gait strategies employed by people with gout may contribute to reduced muscle activity and consequent disuse muscle atrophy [18–20]. Although the current results demonstrated an association between reduced muscle force and pain, even after adjusting for pain scores, it should be noted that pain was assessed over the past week and may not capture cumulative pain over the course of the disease. Several studies assessing foot pain over a longer duration (3 to 20 months) in people with musculoskeletal pathology, have reported associations between foot muscle strength and foot pain [31–33].

The Achilles tendon was the most common site for tophus presence in the foot and ankle of participants, which is consistent with published work using dual-energy computed tomography [29]. The Achilles tendon plays a major role in sagittal plane motion during the gait cycle and works alongside muscular contractions during plantarflexion to assist in the propulsion of body weight [34]. Although construct validity of dynamometry relative to dynamic muscle forces during gait in this population has not been determined, the findings from the current study may suggest that any tophus-associated restrictions in normal function may contribute to the reduced muscular activation during plantarflexion and consequent disuse muscle atrophy.

It is unclear whether the addition of muscle weakness in those with subcutaneous tophi further impairs their ability to carry out functional activities of daily living, including walking [19, 20]. Weakness of foot and ankle musculature has been associated with poor balance and an increased risk of falls in the elderly [35], as well as foot deformity, including hallux valgus - which is common in people with gout [17, 21]. The clinical impact of foot muscle weakness in people with tophaceous gout will be the subject of further research.

The findings from this study should be considered in light of several limitations. The cross-sectional nature of the study limits the ability to determine causation with regard to whether tophus deposition within the musculoskeletal system impairs muscular function and strength, or whether pre-existing abnormalities in musculoskeletal foot function and mechanics predisposes to local urate crystal deposition. The foot pain VAS used in the current study assessed average pain over the past week and may not reflect pain levels experienced earlier in the disease, which may also impact the muscle force assessed during the study. Additionally, physical activity and its relationship with pain, which was not assessed in the current study, may have contributed to the muscle strength deficits. Tophus presence was assessed by clinical examination, which may not be reflective of the true tophus burden in people with gout, evident by imaging studies that have demonstrated subclinical urate deposition in the foot [36]. Static dynamometry may not be reflective of muscle activity during gait. Furthermore, although static dynamometry was chosen based on its clinical utility, it also has limited validity in testing stronger muscle groups such as the plantarflexors due to the possibility of overpowering the examiner [37]. Finally, the examiner could not be blinded to the presence or absence of foot/ankle tophi during muscle testing, and therefore was not blinded to the participant’s group allocation. Although dynamometry is primarily participant-dependent, the possibility of bias related to lack of assessor blinding cannot be excluded.

Longitudinal studies are required to determine the role of tophi in the mechanism for muscle weakness in gout. Additionally, the association between strength deficits and spatiotemporal gait parameters in people with gout may also be assessed. Future work may also employ advanced imaging techniques, such musculoskeletal ultrasound or dual energy computed tomography, to assess the impact of subclinical tophus burden on joint and muscle function in people with gout, both during non-weight bearing and dynamic gait testing. This would greatly advance current understanding of specific factors that may contribute to muscle atrophy as a result of local crystal deposition, including the adoption of pain-avoidance gait strategies, mechanical joint restrictions and impaired musculotendinous function.

## Conclusions

In conclusion, this study has shown that people with gout who have clinically-evident foot or ankle tophi demonstrate significant muscle force deficits during foot plantarflexion, dorsiflexion, inversion and eversion when compared to those without tophi, even after adjusting for recent foot pain, suggesting that other factors may also be driving muscle atrophy in people with gout. This study has also shown that tophus presence specifically at the Achilles tendon is associated with foot and ankle muscle force deficits.

## Abbreviations

1MTP: First metatarsophalangeal joint
BMI: Body mass index
MFPDI: Manchester foot pain and disability index
MSU: Monosodium urate
MTP: Metatarsophalangeal joint
VAS: Visual analogue scale
